# Reduction of charge offset drift using plasma oxidized aluminum in SETs

**DOI:** 10.1038/s41598-020-75282-4

**Published:** 2020-10-26

**Authors:** Yanxue Hong, Ryan Stein, M. D. Stewart, Neil M. Zimmerman, J. M. Pomeroy

**Affiliations:** 1 University of Maryland, College Park, MD, 20742 USA; 2 National Institute of Standards and Technology, Gaithersburg, MD 20899 USA

**Keywords:** Electronic devices, Quantum information, Quantum dots, Quantum physics, Quantum information, Qubits

## Abstract

Aluminum oxide (${\text {AlO}}_x$)-based single-electron transistors (SETs) fabricated in ultra-high vacuum (UHV) chambers using in situ plasma oxidation show excellent stabilities over more than a week, enabling applications as tunnel barriers, capacitor dielectrics or gate insulators in close proximity to qubit devices. Historically, ${\text {AlO}}_x$-based SETs exhibit time instabilities due to charge defect rearrangements and defects in ${\text {AlO}}_x$ often dominate the loss mechanisms in superconducting quantum computation. To characterize the charge offset stability of our ${\text {AlO}}_x$-based devices, we fabricate SETs with sub-1 e charge sensitivity and utilize charge offset drift measurements (measuring voltage shifts in the SET control curve). The charge offset drift ($\Delta {Q_0}$) measured from the plasma oxidized ${\text {AlO}}_x$ SETs in this work is remarkably reduced (best $\Delta {Q_0}=0.13 \, \hbox {e} \, \pm \, 0.01 \, \hbox {e}$ over $\approx 7.6$ days and no observation of $\Delta {Q_0}$ exceeding $1\, \hbox {e}$), compared to the results of conventionally fabricated ${\text {AlO}}_x$ tunnel barriers in previous studies (best $\Delta {Q_0}=0.43 \, \hbox {e} \, \pm \, 0.007 \, \hbox {e}$ over $\approx 9$ days and most $\Delta {Q_0}\ge 1\, \hbox {e}$ within one day). We attribute this improvement primarily to using plasma oxidation, which forms the tunnel barrier with fewer two-level system (TLS) defects, and secondarily to fabricating the devices entirely within a UHV system.

## Introduction

The future of large scale quantum computing depends on successfully merging diverse materials and qubit architectures that each realize different functionalities, i.e., computation, cache, memory or long range transmission. To accomplish this, quantum information must be efficiently transduced between bases, e.g. superconducting and semiconducting qubits, requiring mutually compatible materials and designs. Specifically within those two realms, aluminum and its native oxides (${\text {AlO}}_x$) have enabled great advances, providing simple and reliable tunnel couplings in superconducting circuits^1–3^ and isolation oxides between nanoscale gates for semiconducting qubit control^4,5^. However, superconducting qubits still suffer from unacceptably high relaxation rates, motivating device designs that minimize ${\text {AlO}}_x$ utilization and the electric field density within the oxides (reduced participation factor). Similarly, aluminum implementation in semiconducting devices is most successful the farther the aluminum is from the sensitive region of the device^4,6–8^. In both cases, the amorphous ${\text {AlO}}_x$ formed during thermal oxidation is thought to have a high density of electrically active defects originating from its nonequilibrium structure^9,10^, which interact with the quantum system and create a substantial loss mechanism.

None-the-less, aluminum and ${\text {AlO}}_x$ remain highly desirable choices due to the nearly ideal WKB-like (Wentzel–Kramers–Brillouin) attenuation of states in the tunnel barriers, i.e., minimal tunneling dependence on angular momentum, spin, etc., and their compatibility with nanofabrication techniques. Therefore, efforts to identify and suppress the instabilities of ${\text {AlO}}_x$ are of great significance for enhancing qubit performance. Further, establishing stable aluminum oxides enables expanded use of metal single-electron transistors (SETs), like those used in this study, to be used as surface mounted charge sensors, reducing the density of in-plane circuit elements. These charge sensors could, for example, provide projective spin readout through spin-to-charge conversion techniques already demonstrated^8,11,12^.

SETs are considered to be the world’s most sensitive electrometers, with the capability of detecting the motion of individual electrons or charge instabilities^13–17^. The same sensitivity that enables exquisite readout of a target qubit is also susceptible to any other charge motion within the local environment, e.g., unintentional charge defects. In this study, we use that sensitivity as a probe of whether sufficiently stable charge environments can be realized.

Ideally, the dependence of the SET’s source-drain current $I_s$ on the gate voltage $V_g$ (control curve) will have a periodic behavior with each period corresponding to a 1 e change in the island’s net charge. When the source-drain bias $V_d\approx 0$, this will look like a series of sharp peaks, but at modest bias (temperature), i.e., $0<e{V_d}({k_B}{T_e})<E_C$, the function smooths out similar to a sinusoid, where $E_C$ is the island charging energy, $k_B$ is the Boltzmann constant, *e* is the elementary charge and $T_e$ is the effective electron temperature. Changes in the control curve’s phase indicate uncontrolled changes in the SET’s local electrostatic environment. The time trace of the phase is a sensitive indicator of stability and phase changes are known as charge offset drift, denoted by $\Delta {Q_0}$.

Extensive prior work examining the charge offset stability of various materials showed that metallic SETs incorporating ${\text {AlO}}_x$ tunnel barriers demonstrate severe time instability—random, time-dependent phase fluctuations in the control curve^13–18^. These large, abrupt changes are attributed to two-level system (TLS) defects associated with amorphous ${\text {AlO}}_x$, consistent with similar findings from TLS spectroscopy studies in the context of superconducting qubits^19–21^. In our prior work, we mitigated long-term, macroscopic resistance drift using plasma oxidized ${\text {AlO}}_x$ tunnel barrier devices^22^, a behavior thought to originate from the same bath of defects that causes significant charge offset drift^23,24^. Plasma oxidation, compared to conventional thermal oxidation, incorporates higher oxygen content in the barrier layer at a considerably faster rate, resulting in a much better initial quality^25–27^. To explore whether the macroscopic improvements seen in plasma oxidized ${\text {AlO}}_x$ improve the feasibility for ${\text {AlO}}_x$ use in quantum computing applications, we fabricated metallic SETs with plasma oxidized ${\text {AlO}}_x$ tunnel barriers and measured their long-term charge offset drift. We find these devices exhibit significantly reduced charge offset drift—the best $\Delta {Q_0}=0.13 \, \hbox {e} \, \pm \, 0.01 \, \hbox {e}$ over $\approx 7.6$ days and no observation of $\Delta {Q_0}$ exceeding $1 \, \hbox {e}$, compared with any other previously reported metallic SETs^13,17,18,28^ where the best $\Delta {Q_0}=0.43\, \hbox {e} \, \pm \, 0.007\, \hbox {e}$ over $\approx 9$ days and most $\Delta {Q_0}\ge 1\, \hbox {e}$ within one day. Uncertainties presented in this paper represent standard deviations (SDs) unless otherwise stated. We attribute this improvement to (i) using plasma oxidation to form the tunnel barriers and (ii) fabricating the devices entirely within a system of ultra-high vacuum (UHV) chambers.

## Results

### Device design and characterization

**Figure 1:**
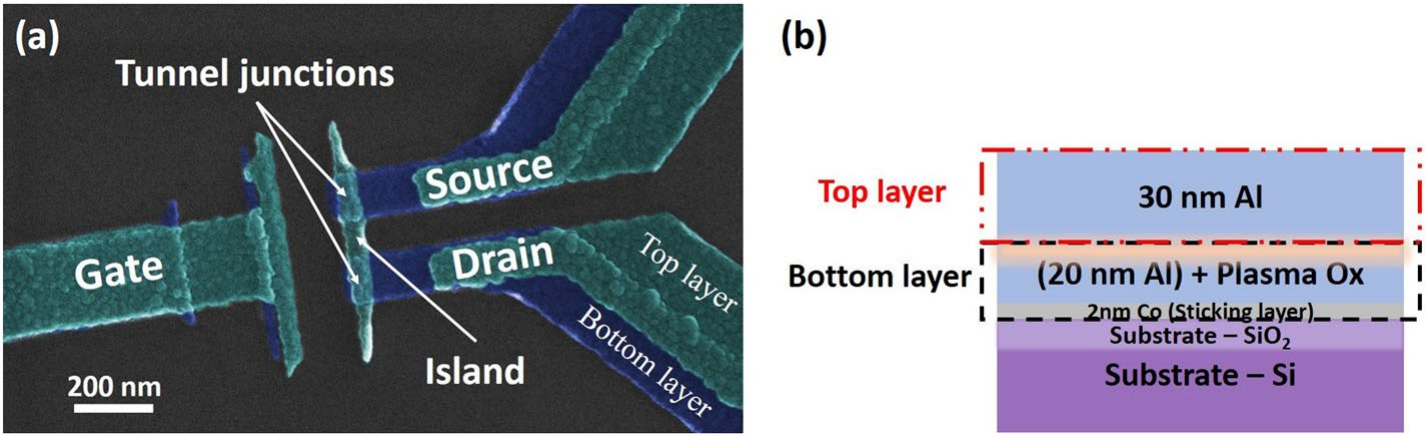
(**a**) False-colored SEM image of an ${\text {Al/AlO}}_x/\text {Al}$ SET identical to the one discussed in this paper. The blue part is the bottom layer with plasma oxide on the surface and the green is the top layer. (**b**) A cross-sectional cartoon through the tunnel junction of the ${\text {Al/AlO}}_x/\text {Al}$ SETs.

The device fine structures are fabricated via double-angle, shadow evaporation^29^ in a system of UHV chambers with a base pressure of $< 10^{-7} \, \hbox {Pa} \, (10^{-9} \, \hbox {Torr})$ equipped with deposition and plasma oxidation capability (see Ref.^22^ for equipment details). Figure 1a shows a scanning electron microscope (SEM) image of the device fine structure with a schematic cross-section through the tunnel junction in Fig. 1b. The blue part is the bottom layer—2 nm cobalt (sticking layer) with 20 nm aluminum deposited at a first angle on the thermally oxidized silicon substrate. The wafer is then transferred to the plasma chamber where the surface of the bottom layer is oxidized using a DC plasma at 21 Pa (160 mTorr) of research grade oxygen with 57 W to 60 W for 7 s. The oxide is then allowed to relax in vacuum for at least 12 h before the top layer is deposited, consistent with prior work^22^ mitigating resistance drift. The ${\text {AlO}}_x$ layer produced under this condition is estimated $\approx 2 \, \hbox {nm}$ thick^22^. Note that although plasma can attack the electron-beam (e-beam) resist used for shadow evaporation, the influence of oxygen plasma on the e-beam pattern at this step is negligible since the resist has been coated by the metal in the first deposition. The wafer is then transferred back to the deposition chamber where 30 nm aluminum is deposited at a second angle for the top layer, as shown in green in Fig. 1a. By controlling the deposition angle, the size of the island and tunnel junctions can be controlled to a precision of $<5 \, \hbox {nm}$ along the direction of rotation. Additionally, the pattern is designed so that, depending on angle, some features do not appear in both layers. During the deposition steps, a liquid nitrogen cryoshroud is kept cold to insure high vacuum. The substrate temperature begins at ambient temperature and is expected to warm somewhat.

When a metallic island is separated from source and drain reservoirs by tunnel junctions, the resulting SET can confine an integer number of electrons on the island. As a result of this charge quantization, the charging energy required to add or remove a single electron from the island is $E_C={e^2}/{C_\Sigma }$, where $C_\Sigma$ is the island’s total capacitance. Coulomb blockade due to discrete electron charges is strongly visible when: i) the tunnel junction conductances $\ll 2e^2/h$ (*h* is the Planck constant), and ii) ${k_B}{T_e}\ll {E_C}$^30^. If the bias ${V_d}\ll {E_C}/e$, then the spacing between single electron energy levels is larger than the bias window $V_d$ and for some values of $V_g$ no energy levels on the island will fall within the bias window. In that case, the source-drain current $I_s$ can be $\approx 0$, known as Coulomb blockade. When a single electron energy level on the island does fall within the source-drain bias window, then electrons can tunnel on and off of the island, producing a source-drain current $I_s$. The maximum of $I_s$ is reached when an energy level is fully within the bias window, producing a conductance peak that may be broadened by bias or temperature. As $V_g$ is swept, different energy levels move through the bias window, producing a periodic current oscillation vs. $V_g$, referred to as Coulomb blockade oscillation (CBO). The oscillation period $\Delta {V_g}$ is determined by the capacitance between the gate and the island, $\Delta {V_g} = e/C_g$. Since the total charge on the metallic island is $\gg 1\, \hbox {e}$, $C_g$ is approximately constant and the charging energy is assumed constant. When the temperature or bias becomes significant compared to the charging energy, these peaks merge into oscillations that are nearly sinusoidal.

To conduct the charge offset drift measurements, the samples are cooled in a cryogen-free dilution refrigerator (DR) with a base temperature of approximately $10 \, \hbox {mK}$. As shown in Fig. 1a, the SET consists of a small conducting island coupled to the source and drain electrodes through two ${\text {Al/AlO}}_x/\text {Al}$ tunnel junctions. The gate electrode is capacitively coupled to the island and manipulates the electrostatic potential via the gate voltage $V_g$, modifying the charge configuration of the island. A small, constant DC bias, $V_d$, is applied on the drain electrode while measuring the current via a transimpedance amplifier on the source electrode, $I_s$. On the time scale of seconds, the standard deviation/typical noise of the current and voltage are $\approx 0.06\, \hbox {pA}$ and $\approx 5 \, \mu \hbox {V}$, respectively. The device’s control curve is observed by measuring $I_s$ vs. $V_g$ applied on the gate electrode. A typical charge offset drift measurement involves repeatedly measuring this control curve every few minutes for about one week, as discussed further below.

**Figure 2:**
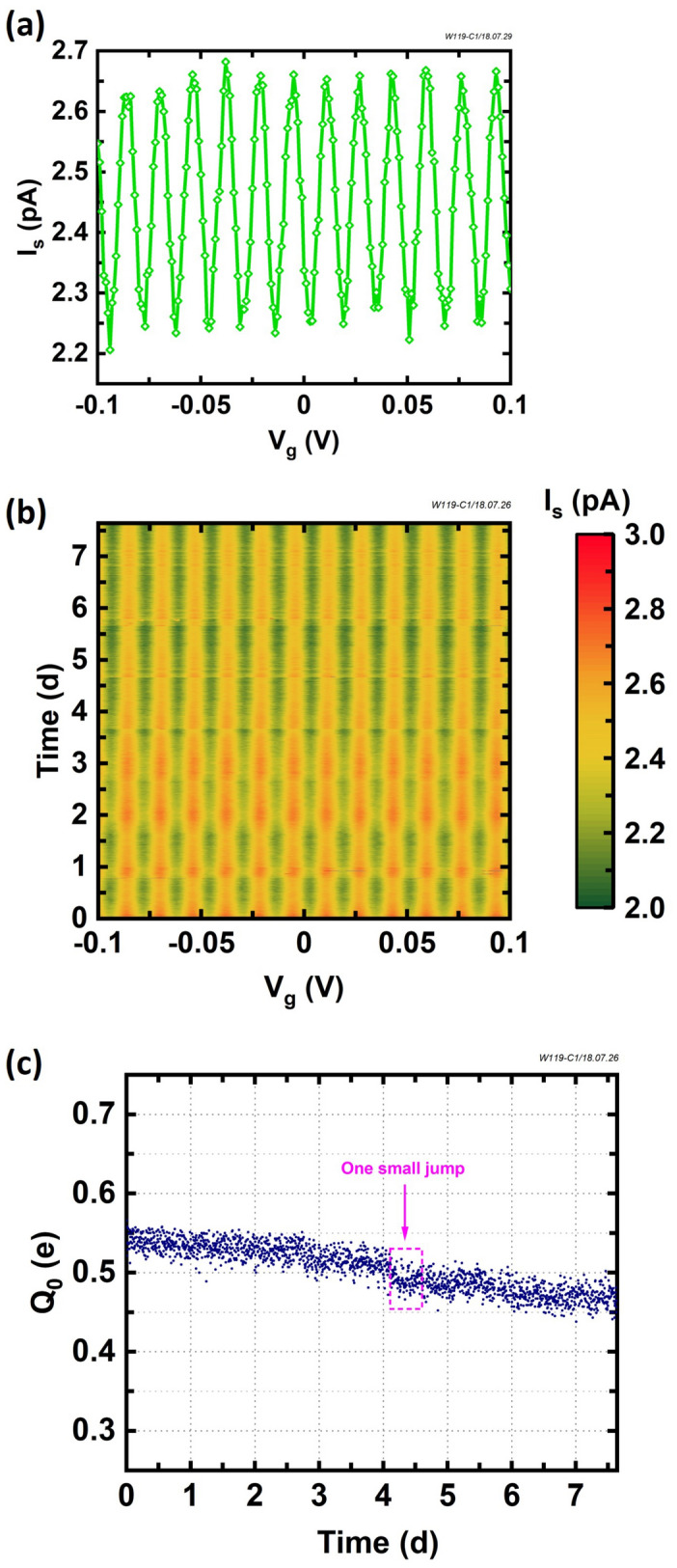
(**a**) A representative $I_s$ vs. $V_g$ CBO from an ${\text {Al/AlO}}_x/\text {Al}$ SET (W119-C1) at $\approx 10 \, \hbox {mK}$ with an applied bias $V_d\approx 0.5 \, \hbox {mV}$ taken from $t\approx 2.3$ d in panel (**b**), a color map of $I_s$ vs. $V_g$ spanning $>1$ week. The vertical stripes indicate that the CBO phase remains stable with time. For this device, $\Delta {V_g}= (16.26\,\pm \,0.04)\, \hbox {mV}$, from which we find a gate capacitance $C_g\approx 9.8$ aF. (**c**) The charge offset, $Q_0$, extracted from the phase in (**b**), as a function of the time. A linear drift of $(-8.2\,\pm \,0.4) \times 10^{-3}\, \hbox {e/d}$ is observed, with one small jump exists from $t\approx 4.1 \, \hbox {d}$ to $t\approx 4.7 \, \hbox {d}$. The total charge offset drift over $\approx 7.6$ days is $\Delta {Q_0}= (0.13\pm 0.011) \, \hbox {e}$.

The devices used in this study and shown in Fig. 1 are designed to exhibit Coulomb blockade behavior at temperatures $<1$ K. Based on the lithographic design and SEM images of similar devices, we estimate the single tunnel junction dimensions are ($47\pm 10$) nm by ($109\pm 10$) nm. Using a parallel plate capacitor model with an ${\text {AlO}}_x$ permittivity of $(10.4\pm 1.1) \cdot \varepsilon _0$ and a thickness of ($2\pm 0.2$) nm, we estimate each junction capacitance to be ($236\pm 65$) aF ($\varepsilon _0$ is the vacuum permittivity). A gate capacitance of ($6.9\pm 3.1$) aF was calculated by modelling a gate of ($560\pm 50$) nm $\times$ ($80\pm 10$) nm $\times$ ($40\pm 10$) nm (length $\times$ width $\times$ height) and an island of ($560\pm 50$) nm $\times$ ($47\pm 10$) nm $\times$ ($40\pm 10$) nm with a separation of ($145\pm 10$) nm on a ${\text {SiO}}_2$/Si substrate using the capacitance solver FastCap and a ${\text {SiO}}_2$ permittivity^31^ of $3.9\cdot \varepsilon _0$. Therefore, a CBO period of ($23.2\pm 10.4$) mV is expected on these devices. Adding up these capacitance gives an expected charging energy, $E_C/k_B= (3.9\pm 1.1) \, \hbox {K}$.

### Charge offset drift measurement

In prior studies of ${\text {Al/AlO}}_x/\text {Al}$ SETs, the local electrostatic environment of the island would often change with time randomly, which appears as the phase of the CBO fluctuating with time. This time instability, referred to as charge offset drift, has been an issue in metallic SET devices for a long time. Many factors contribute to the vulnerable electrostatic environment of the island, including unintentional charge defects from multiple fabrication processes, temporal or thermal material relaxation, circuit noise, etc.

**Figure 3:**
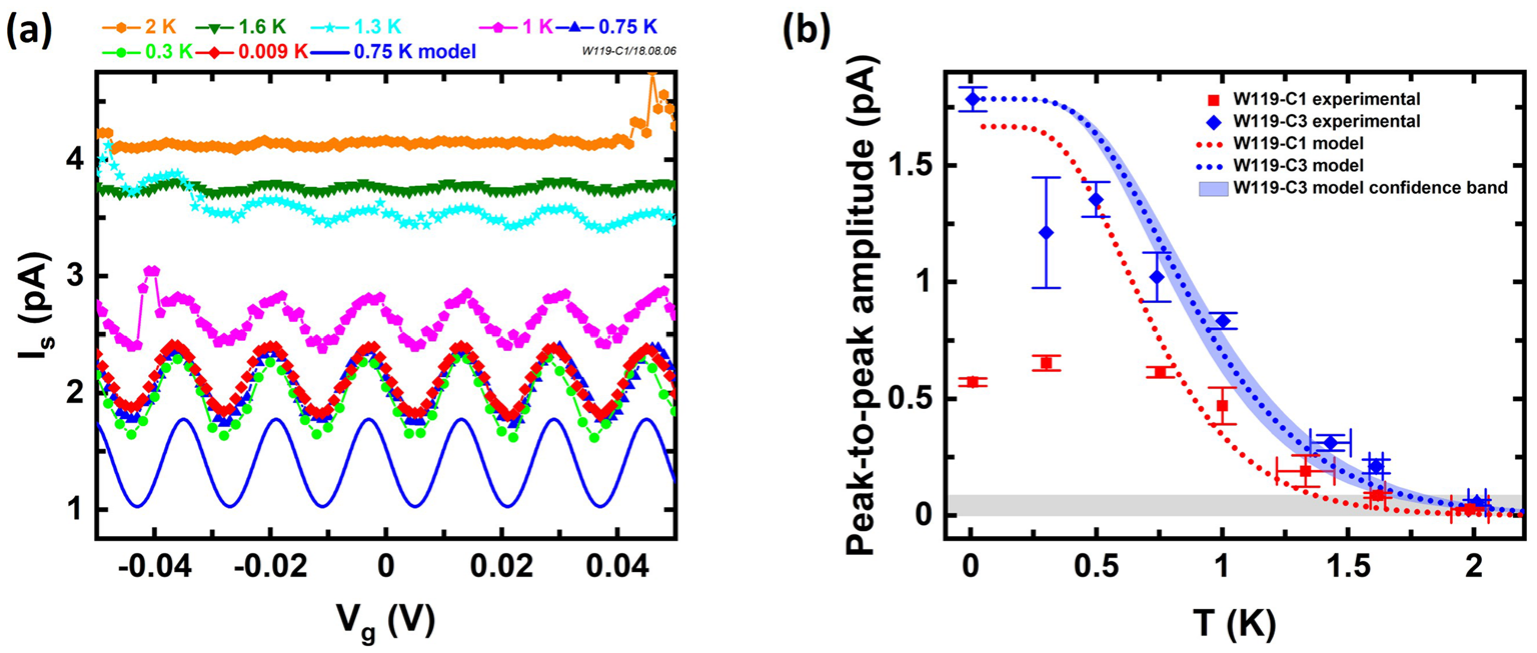
(**a**) CBO on the ${\text {Al/AlO}}_x/\text {Al}$ SET (W119-C1) at varying temperatures. As expected, the oscillations die down with increasing temperature and vanish for $T > 1.3 \, \hbox {K}$ when the oscillations drops below the noise—gray shaded region in (**b**). The blue solid line at the bottom represents the model CBO curve at 0.75 K as discussed in the main text. (**b**) The symbols show CBO peak-to-peak amplitudes extracted from the oscillations like those in (**a**) vs. temperature. Red squares (blue diamonds) are extracted from W119-C1 in (**a**) (W119-C3 in Fig. S1**d** of the supplementary information). The two data points near 2 K are found by fitting a sine function to the CBO to suppress noise and the fitting errors are represented by the vertical error bars. All other data points are peak-to-peak amplitudes found by the average of adjacent peak-to-valley values from the CBO and the vertical error bars represent the standard deviation of all peak-to-valley values at that temperature. The horizontal error bars represent temperature fluctuations within $\pm \,3$ min of the log time. Temperature errors $<20 \, \hbox {mK}$ are not shown. The red (blue) dotted line represents the peak-to-peak trend from the model when $E_C/k_B=4.5 \, \hbox {K} \, (5.6 \, \hbox {K})$ for $R_\infty = 150 \; \mathrm{M}\Omega \, (140 \; \mathrm{M}\Omega )$. The blue shaded region shows the model variation when $C_\Sigma$ varies such that $E_C/k_B$ varies over a range of $\pm \,0.3 \, \hbox {K}$, centered around the best-fit value. The gray shaded region at the bottom indicates the measurement noise.

Figure 2a illustrates an example CBO from an ${\text {Al/AlO}}_x/\text {Al}$ SET (W119-C1) at the base temperature of $\approx 10 \, \hbox {mK}$, with oscillations from each single electron conductance peak moving through the bias window as $V_g$ is swept. In this case, the bias voltage $V_d\approx 0.5 \, \hbox {mV}$ is applied on the drain electrode, with the current measured on the source, and the current oscillates sinusoidally. The nonideal broadening is believed to be due to a noise-induced high $T_e$, which prevents complete Coulomb blockade, discussed further below. The nonzero current offset observed in the CBO is attributed partially to the merged peaks and an imperfect zero on the current preamplifier. For this device, the oscillation period $\Delta {V_g}= (16.26\pm 0.04) \, \hbox {mV}$, from which the gate capacitance $C_g=e/\Delta {V_g}\approx 9.8$ aF, is in agreement with the design estimate of ($6.9\pm 3.1$) aF.

To evaluate the charge offset stability in our plasma oxidized SETs, the CBO is repeatedly measured every five minutes over many days for device W119-C1. The result of this long-term repetitive measurement is shown in Fig. 2b. For each $V_g$ sweep, the value on the y-axis is the time when that sweep ends. The color scale on the *z*-axis represents the $I_s$ value measured from the source electrode. The high and low current values form vertical stripes in time, which clearly show that the CBO phase in our ${\text {Al/AlO}}_x/\text {Al}$ SET remains quite stable without sudden shifts over the course of more than 7 days. Compared to the results from Al SET devices with thermally oxidized ${\text {AlO}}_x$ tunnel barriers^13,17,18,28^, this device exhibits much improved charge offset stability.

To numerically evaluate the stability, we extract the charge offset, $Q_0$, from the repeated sweeps. Here we use two methods for the data compared in this paper and the two methods are consistent within 10 %, verified by Hu et al.^32^ If the source-drain current oscillates nearly sinusoidally as a function of the gate voltage, the measured $I_s$ vs. $V_g$ is fit to:

1 $$\begin{aligned} I_s^t(V_g) = A_s\cdot \sin [2\pi (V_g/\Delta {V_g} + Q_0^t/e)] + I_0 \end{aligned}$$

where $A_s$ is the oscillation amplitude, $Q_0^t$ is the phase for a given *t* and $I_0$ is a nonideal offset. For each line in Fig. 2b, the measured current data is fit to Eq. 1. Then the charge offset as a function of each sweep, $Q_0(t)$ as a set of $Q_0^t$, is plotted as a function of time in Fig. 2c. These data do not exhibit the dramatic, abrupt jumps characteristic seen in devices of prior work (one small jump exists from $t\approx 4.1 \, \hbox {d}$ to $t\approx 4.7 \, \hbox {d}$), but do show a slow, linear drift of $(-8.1\pm 0.6) \times 10^{-3}\, \hbox {e/d}$. The total charge offset drift over $\approx 7.6$ days is $\Delta {Q_0}= (0.13\pm 0.011) \, \hbox {e}$, where $\Delta {Q_0}$ is defined as the full range of $Q_0$ values measured and the uncertainty is calculated as the standard deviation of 100 data points in a stable range. For previously reported metallic SETs with thermally oxidized ${\text {Al/AlO}}_x/\text {Al}$ tunnel junctions, most $\Delta {Q_0}$ are much greater than $1\, \hbox {e}$ and show many abrupt jumps^13,17,18,28^.

When the SET control curve displays sharper peaks and cannot be well fit sinusoidally, individual peaks of the CBO are fit to a Gaussian to locate the peak’s center position:

2 $$\begin{aligned} I_s(V_g) = I_0 + A_g\cdot \exp [-((V_g - V_c)/V_w)^2] \end{aligned}$$

Here, $A_g$, $V_c$ and $V_w$ denote the area/height parameter, center position and full width at half maximum (FWHM) of each Gaussian peak, respectively. In this case, $Q_0(t)=e\cdot \bmod [V_c(t)/\Delta {V_g}]$, where $\Delta {V_g}$ is the gate voltage difference between two adjacent peaks. Data from another device (W119-C3) is shown in the supplementary information where $Q_0(t)$ is calculated in this way. That device shows one abrupt jump of $\delta {Q_0}\approx 0.07\, \hbox {e}$ after $t\approx 5.6$ d of measurement, but is otherwise stable with a linear drift of $(21\pm 1)\times 10^{-3}\, \hbox {e/d}$. In that device, we find the $\Delta {Q_0}$ over $\approx 7.6$ days is ($0.30\pm 0.014$) e. A summary of results from three plasma oxidized ${\text {Al/AlO}}_x/\text {Al}$ devices fabricated in this work, three thermally oxidized ${\text {Al/AlO}}_x/\text {Al}$ devices presented in Ref.^18^, and the best known $\Delta {Q_0}$ result from an all-silicon device with no metals published in Ref.^33^, which is thought to have less TLS defects than devices containing ${\text {AlO}}_x$, are shown in Table 1. The measurement duration ‘$t_{\mathrm{meas}}$’, ‘jumps’ and $\Delta {Q_0}$ from the longest single cooldown of each device are calculated and compared. The uncertainty of $\Delta {Q_0}$ in each device (not relevant on the two devices drifting more than 1 e) is defined as the standard deviation of 100 data points in one stable range, which indicates the measurement stability. For devices fabricated in this work, W119-C3 has the same device geometry as W119-C1 (the one discussed above), while W119-T1-2 has an alternative “in-line” geometry (shown in the supplementary information), but all of them exhibit extended periods ($> 1.5$ days) without jumps. Note that the charge offset drift measurements of W119-T1-2 were often interrupted and stressed by other measurements (see supplementary information for details). This device correspondingly exhibits jumps after measurement breaks and larger $\Delta {Q_0}$ uncertainty, but the charge offset stability in each continuous interval remains stable. As summarized in Table 1, all our plasma oxidized ${\text {Al/AlO}}_x/\text {Al}$ devices are much more stable than the historic thermally oxidized ‘NIST-G’ and ‘NIST-B’ devices in Ref.^18^, while they are comparable to the ‘PTB’ device, which is thought to be the best metallic ${\text {Al/AlO}}_x/\text {Al}$ device in Ref.^18^. The nonmetallic, silicon SOI (silicon-on-insulator) device from Ref.^33^ is included for reference as one of the best results from an extended charge offset stability measurement. We systematically find our metallic devices are much more stable, with no evidence in any measurements of the gross, erratic charge offset seen in the historic metallic devices.

**Table 1:** Comparison of charge offset stabilities for several ${\text {Al/AlO}}_x/\text {Al}$ devices used in this study and available in the literature, with a silicon SOI device as a high quality benchmark in the last row.

Device	$t_{\mathrm{meas}}$ (d)	Jumps	$\Delta Q_0$ (e)
W119-C1	7.6	1	$0.13\pm 0.011$
W119-C3	7.6	1	$0.30\pm 0.014$
W119-T1-2	3.9	2^a^	$0.68\pm 0.038$
NIST-G (Ref.^18^—Fig. 3)	18.8	> 100^b^	$\ge 1$
PTB (Ref.^18^—Fig. 4)	9.0	7^c^	$0.43\pm 0.007$
NIST-B (Ref.^18^—Fig. 5)	7.5	> 50^b^	$\ge 1$
SOI Si (Ref.^33^—Fig. 7)	7.9	0	$0.03\pm 0.003$

W119-C1 charge offset drift data are shown in the main text, other new data presented in this study are in the supplementary information. $t_{\mathrm{meas}}$ is the total span of charge offset drift data, which includes break in some cases, a ‘jump’ occurs when the 100 pt running standard deviation increases by a factor of three, ‘$\Delta Q_0$’ is defined as the full range of the $Q_0$ values measured.

^a^Non-contiguous measurements with multi-hour breaks;^b^Main method doesn’t apply and number of jumps is roughly estimated by times of $Q_0$ change $>0.2\, \hbox {e}$.^c^5 out of 7 jumps are correlated with liquid helium transfers.

### Temperature dependence measurement

Finally, we use the temperature dependence of the current lineshape to determine the total capacitance $C_\Sigma$ and charging energy $E_C$ for two devices in comparison with the design values. The absence of strong Coulomb blockade in W119-C1, as shown in Fig. 2, prevents estimation of $C_\Sigma$ and $E_C$ using a Coulomb diamond measurement. As mentioned earlier, strong Coulomb blockade behavior can only be observed when ${k_B}{T_e}\ll {E_C}$. As the bath temperature increases, the source/drain reservoirs broaden and the single electron conductance peaks will be thermally broadened^34^. An individual current peak in this regime is described as^30,34,35^:

3 $$\begin{aligned} I\approx \frac{1}{2}I_{\infty }\cdot \cosh ^{-2}\left[ \frac{\alpha e (V_g-V_c)}{2.5{k_B}{T_e}}\right] \end{aligned}$$

where $I_{\infty }=V_d/R_{\infty }$ is the reference current characterized by the bias voltage, $R_{\infty }$ the device resistance outside of blockade ($V_d \gg E_C/e$), and $\alpha = C_g/C_\Sigma$ is the lever arm. Therefore, as the bath temperature (*T*) increases from the base temperature ($\approx 10$ mK), the current peaks spaced by $\Delta {V_g}$ thermally broaden and eventually blend together. Experimentally, the CBO is typically only visible when ${k_B}{T_e}<0.3E_C$^30^. Figure 3a shows CBO sweeps as a function of the temperature from W119-C1. As expected, the oscillations die down with increasing temperature and vanish at temperatures above $>1.3$ K, when the oscillation is lost in the noise. The blue solid line at the bottom in Fig. 3a shows the model CBO curve calculated from an array of 25 current peaks (Eq. 3) that span $V_g=0$ spaced by $\Delta {V_g}$ based on the extracted $C_g$ and $\Delta V_g$ values as listed above, ${T_e}=0.75 \, \hbox {K}$, $R_{\infty } = (133 \pm 20)\; \mathrm{M}\Omega$ (taken from separate $I_s$ vs. $V_d$ measurements) and $C_\Sigma$ adjusted to match the temperature dependence below. Note that it is important to include sufficient number of peaks outside the window to capture tails of peaks. The peak-to-peak amplitude of the CBO vs. temperature is shown in Fig. 3b where the red squares (blue diamonds) are from W119-C1 (W119-C3) in Fig. 3a (Fig. S1d in supplementary information). The red (blue) dotted line shows the peak-to-peak amplitudes taken from model curves like that shown in Fig. 3a, where $C_\Sigma =410$ aF (329 aF) is adjusted to best capture the experimental trend while using all other experimentally determined quantities. The blue shaded region around the blue dotted line shows the peak-to-peak amplitude range for W119-C3 corresponding to $E_C/k_B$ varying over a range of $\pm \,0.3 \, \hbox {K}$, centered around the best-fit value, where $R_{\infty } = (145 \pm 20)\; \mathrm{M}\Omega$. The experimental values and the model values agree well at temperatures $\ge 0.75 \, \hbox {K}$. The discrepancy at temperatures below 0.75 K implies that the electron temperature for W119-C1 is around $(0.5{-}0.75) \, \hbox {K}$. The gray shaded region at the bottom indicates the limit given by the current noise. The oscillations are not visible when the temperature is $>1.3 \, \hbox {K} \, (1.6 \, \hbox {K})$, which also agrees with Ref.^30^ that oscillations are not visible when ${k_B}T\ge 0.3E_C$. The good agreements between the lines and the data allow us to constrain the uncertainty of ${E_C}/{k_B}$ to within about $\pm \,0.3 \, \hbox {K}$ and the best-fit ${E_C}/{k_B}$ values agree well with the design values for these devices given above. Taken as a whole, these measurements indicate the devices were realized as designed and are functioning in the single electron regime consistent with the accepted theory.

## Discussion

We now discuss the microscopics that can explain improved charge offset stability. The charge offset drift is thought to be caused by a broad distribution of electrically active defects associated with amorphous ${\text {AlO}}_x$ tunnel barriers or gate dielectrics fabricated by conventional thermal oxidation^13,17,36^. Among these defects, TLSs originating from the initial nonequilibrium structure of ${\text {AlO}}_x$ are quite common, which is also the predominant decoherence source in superconducting qubits^37^. The exact location of the TLSs in the device that adversely affects the charge offset noise remains uncertain, specifically on whether they reside in the tunnel barriers, in the substrate material, or both^38–40^. Additionally, in a recent paper^32^ and unpublished data (private communication with M. D. Stewart, Jr. research group), $\Delta {Q_0}$ reductions were observed by adding a poly-Si top gate on bulk silicon SETs and replacing bulk Si with SOI in SETs using ${\text {AlO}}_x/\text {Al}$ metal gates. These results imply that the ${\text {AlO}}_x$ instability is not the only factor affecting the magnitude of the charge noise, but that deliberate device design can also mitigate defect interactions with quantum dots.

Devices fabricated in the present work did not utilize any geometries expected to mitigate charge offset drift. Therefore, we attribute the significant reduction of charge offset drift to i) the better initial oxide quality achieved in the tunnel barrier using plasma oxidation, and ii) the complete processing within a UHV environment. Firstly, previous studies^25–27^ have shown that in plasma oxidation, the Al layer can incorporate much more oxygen in a much shorter time and the oxide is much closer to stoichiometric ${\text {Al}}_2{\text {O}}_3$ than in conventional thermal oxidation. In plasma oxidation, the neutrally charged, low energy ${\text {O}}^{\star }$ free radicals are the dominant reactive species that accelerate the oxidation process^25^, and our plasma is tuned to optimize ${\text {O}}^{\star }$ production. Therefore, a smaller number of unoxidized Al defects are expected in ${\text {AlO}}_x$ tunnel barriers produced by plasma oxidation relative to those formed by thermal oxidation. We have previously shown reduced long-term resistance drift on ${\text {AlO}}_x$ tunnel barrier devices^22^, consistent with this picture. Secondly, UHV conditions for contamination control are well established to reduce impurity concentrations^41^ and improve surface smoothness^42^ in the thin Al film deposition, as well as limiting uncontrolled oxide formation in the as-deposited Al layer before deliberate oxygen plasma treatment and reducing structural defects in the tunnel barriers. We propose that plasma oxidation is more likely to be the dominant underlying cause for the significant $\Delta {Q_0}$ reduction, but either mechanism could be significant.

We also implemented plasma oxidized ${\text {AlO}}_x$ tunnel barriers in SETs fabricated from other materials and measured their long-term charge offset drift. Qualitatively, we see improvements in the charge offset drift compared to the historical thermal oxide data shown in Table 1, but it is not as dramatic as in these aluminum only devices. Example data measured in ${\text {Co/AlO}}_x/\hbox {Co}$ devices are shown in the supplementary information, however, the process optimization and data are far less complete for these devices.

Finally, the stability of these devices suggests an opportunity to evaluate ${\text {Al/AlO}}_x/\text {Al}$ SETs as charge sensors for MOS (metal-oxide-semiconductor) quantum dot based charge qubits or spin-to-charge conversion in spin qubits. In this application, an abrupt change in $I_s$, corresponding to a 1 e change on a capacitively-coupled qubit, is used to detect a change in qubit charge configuration. The qubit is typically configured such that a charge transition corresponds to a projective readout of the qubit. However, operationally, the charge sensor is often operated in a constant feedback mode, where instead of change in current, an abrupt change in the gate voltage (charge offset) needed to maintain a constant charge sensor current indicates a charge transition. In order to hypothetically consider W119-C1 as a charge sensor, we set the criterion that the change in the control voltage (charge offset) due to a charge transition must exceed 5 SD (99.97 % readout confidence) of the noise for a period long compared to a measurement. For W119-C1, a SD of $Q_0$ over a 10-hour period is 0.011 e (also the uncertainty in Table 1), corresponding to a SD in gate voltage $\approx 0.18 \, \hbox {mV}$ ($\Delta {V_g}\approx 16 \, \hbox {mV}$). Therefore, as long as ${C_c}/{C_{\Sigma \_\mathrm{MOS}}}>5\mathrm{SD}$ or ${C_c}/{C_{\Sigma \_\mathrm{MOS}}}>0.055$, where $C_c$ is the coupling capacitance between the MOS quantum dot and the SET charge sensor and $C_{\Sigma \_\mathrm{MOS}}$ is the total capacitance of the quantum dot, the induced charge change on the charge sensor can be distinguished from background instability with 99.97 % confidence. For a typical silicon MOS quantum dot, the total capacitance is $\approx 50$ aF and requires ${C_c}>2.8$ aF, which is quite small compared to typical coupling capacitances (for example $\approx 20$ aF in Ref.^43^). Given the long term stability seen in these devices, this hypothetical operation could be expected to persist for periods $>1$ week without any retuning events.

In summary, we fabricated novel ${\text {Al/AlO}}_x/\text {Al}$ SET devices incorporating plasma oxidized ${\text {Al/AlO}}_x/\text {Al}$ tunnel barriers in a UHV system. Much improved charge offset stabilities are observed in these devices in multi-day measurements, in contrary to the large charge offset drift measured from historical devices using typical, thermally oxidized ${\text {Al/AlO}}_x/\text {Al}$ tunnel barriers. Numerical results demonstrate this improvement quantitatively. Two factors combine to suppress the TLS defects in the ${\text {Al/AlO}}_x/\text {Al}$ tunnel barriers: i) Plasma oxidation, which is a more efficient oxide process and can provide better initial oxide quality, and ii) the UHV environment, which can drastically reduce contamination in the entire fabrication process and preserve the quality of the formed ${\text {Al/AlO}}_x/\text {Al}$ tunnel barriers. Future experiments using thermal oxidation in the UHV environment could separate the impacts of these two possibilities. We speculate that there is some overlap between i) the class of slow defects (time scale: hours or days) that generate slow charge offset drift and ii) the class of faster defects (time scale: microseconds) that lead to decoherence from relaxation or dephasing. For this reason, success in suppressing time instabilities in ${\text {AlO}}_x$ may pave the way to reducing some decoherence sources associated with ${\text {AlO}}_x$^9,10^ and enabling expanded implementation within quantum computation systems.

## Methods

In order to reduce the fabrication time and increase volume, the macroscopic contact electrodes and interconnects are fabricated first by wafer-scale photolithography, sputter deposition of 10 nm titanium and 50 nm gold, and a subsequent lift-off process. The device fine structures are fabricated by double angle, shadow evaporation techniques^29^ using polymethyl methacrylate (PMMA) and methyl methacrylate (MMA) double-layer stencil masks patterned by high-resolution e-beam lithography. All of the steps leading up to deposition of the fine structure are performed *ex situ* in a nanofabrication facility. Once the large-scale fanout and nano-scale lithographic stencils are complete, the wafers are loaded into a system of UHV chambers with a base pressure of $< 10^{-7} \, \hbox {Pa} \, (10^{-9} \, \hbox {Torr})$ equipped with deposition and plasma oxidation capability for the formation of fine structures. By combining e-beam lithography with photolithography, the total lithographic exposure time is $< 1 \, \hbox {h}$ for a 75 mm wafer with 100 devices.

## Supplementary information

Supplementary Information.

## Data Availability

The data that support the findings of this study are available from the corresponding author upon reasonable request.
