# Supplementary material for: Reduction of charge offset drift using plasma oxidized aluminum in SETs
Source: Sci Rep. 2020 Oct 26;10:18216. doi: 10.1038/s41598-020-75282-4 (PMC7588434; doi:10.1038/s41598-020-75282-4)
Supplement: Supplementary file 1 — Supplementary Information. [file 41598_2020_75282_MOESM1_ESM.pdf]

Reduction of Charge Offset Drift Using Plasma Oxidized Aluminum in SETs

Yanxue Hong[1,2], Ryan Stein[1,2], M. D. Stewart, Jr[2], Neil M. Zimmerman[2], J. M. Pomeroy[2]

[1]*University of Maryland, College Park, Maryland, 20742, USA.*

[2]*National Institute of Standards and Technology,*
Gaithersburg, Maryland, 20899, USA.

Supplementary Information

I. Long-term charge offset drift measured on W119-C3

We measured long-term charge offset drift on two plasma oxidized Al/AlO_x/Al SETs at the same time. In addition to the one shown in the main text (W119-C1), the results from the other SET (W119-C3) are shown in Fig. S1. From the CBO shown in Fig. S1 (a), we can see that this device exhibits larger oscillation period (ΔV_g = 17.9 mV ± 0.5 mV) but narrower peak width (FWHM = 6.3 mV ± 0.3 mV for a typical peak at base temperature) compared to the device shown in the main text (ΔV_g = 16.26 mV ± 0.04 mV and FWHM = 10.7 mV ± 0.7 mV for a typical peak at base temperature). This results in a smaller linewidth/period ratio, which suggests lower noise/temperature than W119-C1. Again, the nonzero current offset (negative in this case) observed in the CBO is attributed to an imperfect zero on the current preamplifier. Two different preamplifiers were used on each device and had different current offsets. The long-term repetitive CBO in Fig. S1 (b) and the charge offset (Q_0) data in Fig. S1 (c) display faster linear drift of (21 ± 1) x 10^3 e/d in this device and an abrupt phase shift at t ≈ 5.6 d. This Al/AlO_x/Al SET is also very stable, e.g., charge offset drift ΔQ_0 = (0.30 ± 0.014) e over ≈ 7.6 days, compared to previous thermally oxidized Al/AlO_x/Al SETs [Ref. 14, 18-19, 29 in the main text]. Finally, Fig. S1(d) is the experimental CBO vs. temperature data with a model curve at 0.75 K on this device showing the oscillations dying down with rising temperature and vanishing at T > 1.6 K, which verifies the Coulomb blockade of SET. As discussed in the main text, we estimate the charging energy of this device to be E_C/k_B = 5.6 K.

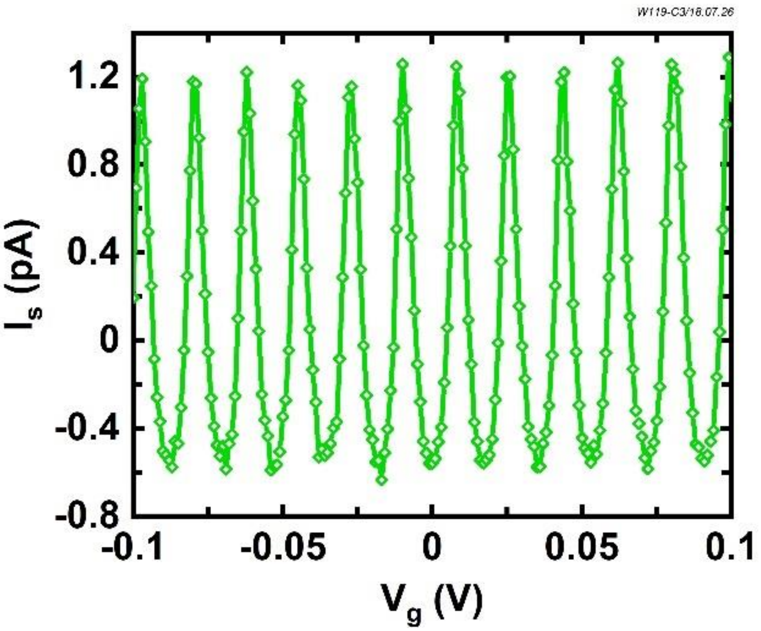
W119-C3/18.07.26
1.2
0.8
0.4
0
-0.4
-0.8
I_s (pA)
-0.1
-0.05
0
0.05
0.1
V_g (V)

(a)

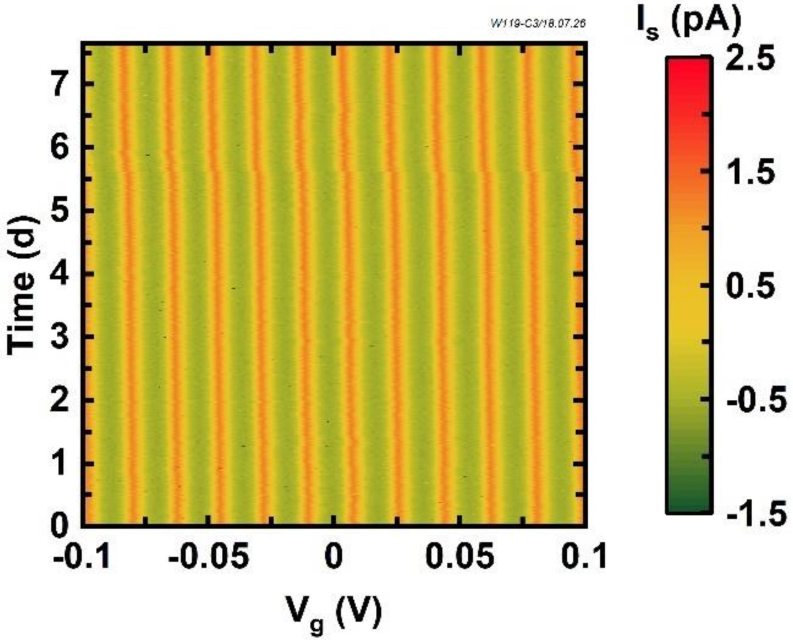
W119-C3/18.07.26
I_s (pA)
2.5
1.5
0.5
-0.5
-1.5
7
6
5
4
3
2
1
0
Time (d)
-0.1
-0.05
0
0.05
0.1
V_g (V)

(b)

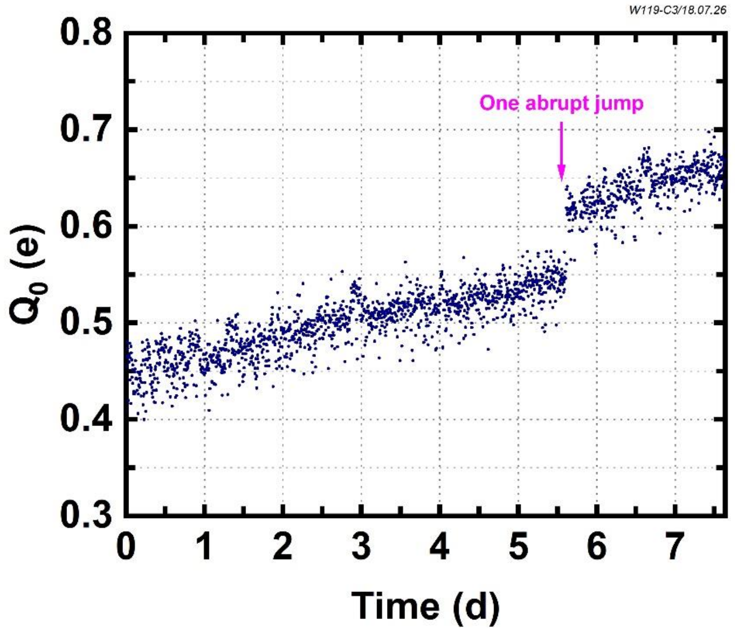

(c)

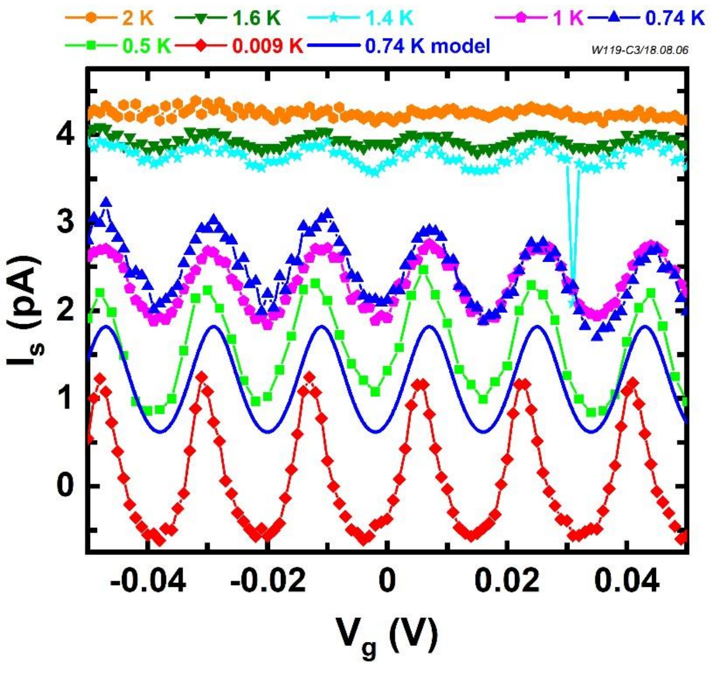

(d)

Fig. S1 Measurement results on W119-C3, another plasma oxidized $Al/AlO_x/Al$ SET. (a) An example CBO at ≈ 10 mK with an applied bias V_d ≈ 0.5 mV taken from t ≈ 5 h from panel (b). This device exhibits larger oscillation period (ΔV_g = 18.3 mV ± 0.3 mV) but narrower peak width (FWHM = 6.3 mV ± 0.3 mV from a typical peak at base temperature) compared to the device shown in the main text (ΔV_g = 16.26 mV ± 0.04 mV and FWHM = 10.7 mV ± 0.7 mV from a typical peak at base temperature). (b) Long-term repeating CBO taken over one week as a function of time. Red and green stripes represent peak and valley of CBO, respectively. (c) Extracted charge offset, Q_0, as a function of time using Guassian method.

This device displays a linear drift of (21 ± 1) x 10^{-3} e/d and an abrupt phase shift at t ≈ 5.6 d. ΔQ_0 = (0.30 ± 0.014) e over ≈ 7.6 days. (d) Measured CBO vs. temperature; the CBO oscillations die down with rising temperature and vanish at T > 1.6 K. The blue solid line represents the model CBO curve at 0.74 K.

II. Charge offset drift measured on $Al/AlO_x/Al$ SET devices of an "inline" geometry

We also fabricated devices with different geometries. Fig. S2(a) shows the SEM image of two SET islands parallel to each other. The two SETs are reflections of each other with an "in-line" arrangement. Shown in Fig. S2(b) and (c) are the charge offset drift measured on an in-line $Al/AlO_x/Al$ SET (W119-T1-2), which was interrupted by other experiments several different times, represented by the breaks on the time scale. Jumps in the current offset value are due to other circuit nonidealities in the low bias regime. Due to the stronger gate capacitance resulted from this geometry, this device exhibits a smaller oscillation period (ΔV_g = 9.8 mV ± 0.5 mV) as expected. Total charge offset drift, ΔQ_0 = (0.68 ± 0.038) e over ≈ 3.9 days with multi-hour breaks. We can see that the oscillation phase and current level fluctuate between measurement intervals, but are stable within an interval. In the middle period of the measurements, there is a 180° phase shift from the beginning, but the phase stays stable for more than two days regardless of the interruptions.

(a)

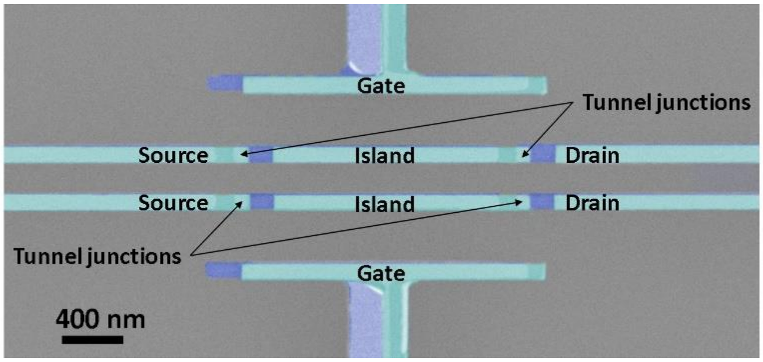

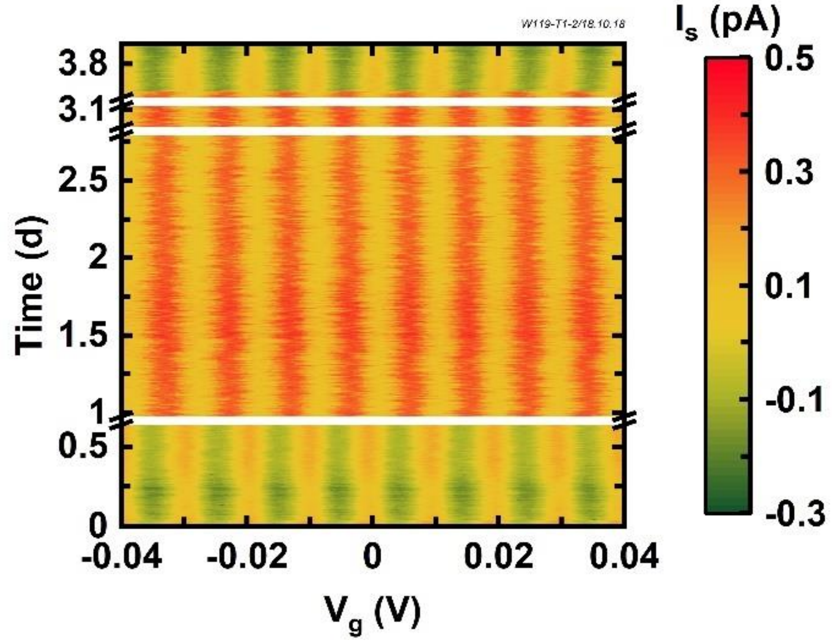

(b)

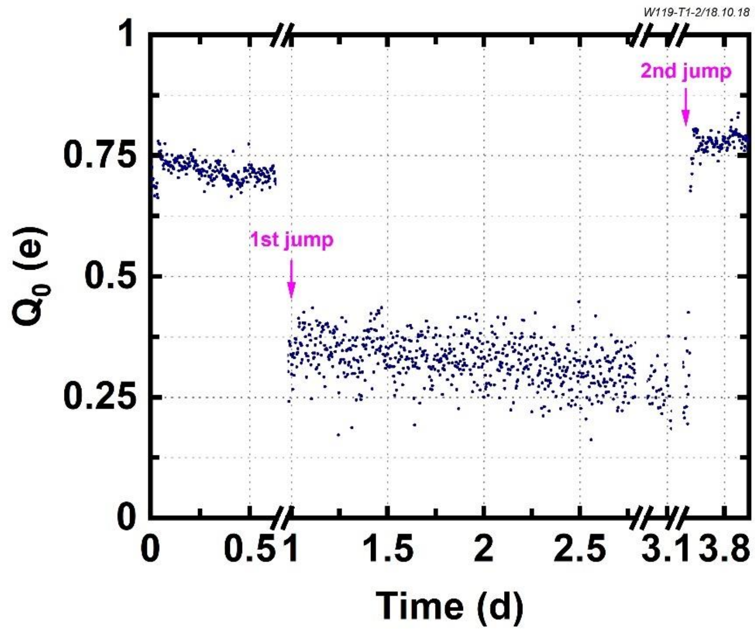

(c)

Fig. S2 (a) False-colored SEM image of in-line SET devices. The blue part represents the bottom layer with plasma oxide on the surface and the green is the top layer. (b) and (c) show the measurement results on an in-line Al/AlO_x/Al SET (W119-T1-2), which was interrupted by other experiments several different times, represented by the breaks on the time scale. (b) Long-term repetitive CBO taken over ≈ 3.9 days as a function of time (ΔV_g = 9.8 mV ± 0.5 mV). Jumps in the current offset value are due to other circuit nonidealities in the low bias regime. (c) Extracted charge offset, Q_0, as a function of time using Sine

method. ΔQ_0 = (0.68 ± 0.038) e over ≈ 3.9 days with multi-hour breaks. Breaks in the horizontal axes correspond to interruptions from performing other measurements.

III. Charge offset drift measured on plasma oxidized SETs made using Co/AlO_x/Co

We also implemented plasma oxidized AlO_x tunnel barriers into Co/AlO_x/Co devices. Fig. S3(a) and (b) show the charge offset drift measured from a Co/AlO_x/Co SET (W118-I4) having the same device geometry but different layer structures as W119-C1 in the main text. Compared to the Al/AlO_x/Al devices with the same geometry, this device exhibits a larger oscillation period (ΔV_g = 22.81 mV ± 0.08 mV) which indicates weaker gate coupling. There are more jumps over the measurement course than above, but are also long periods where the phase is stable and shows very small linear drift (0.013 e/d ± 0.0002 e/d). Total charge offset drift, ΔQ_0 = (0.64 ± 0.007) e. Very little charge offset drift data on Co/AlO_x/Co SETs is present in the literature, but the Q_0 shown here is still much better than the previous thermal oxidized Al/AlO_x/Al SETs [Ref. 14, 18-19, 29 in the main text].

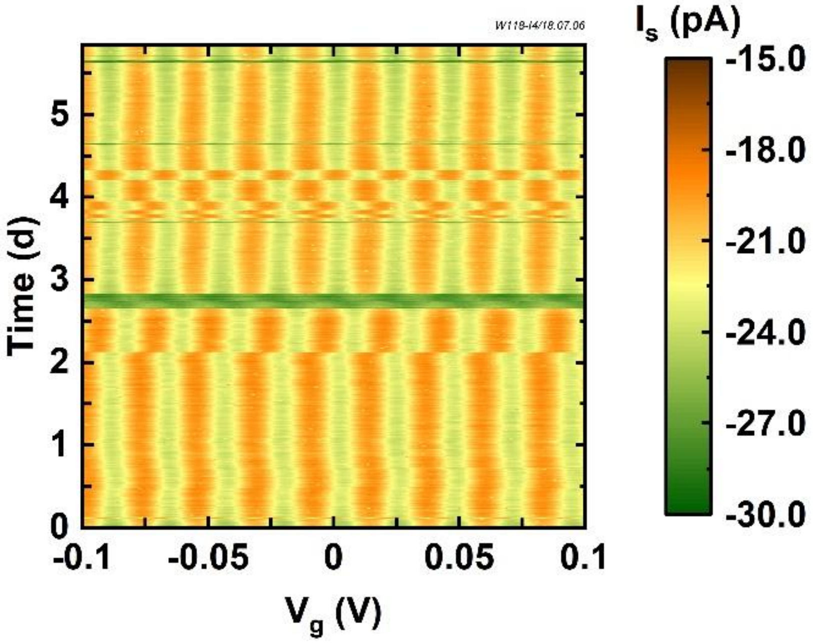

(a)

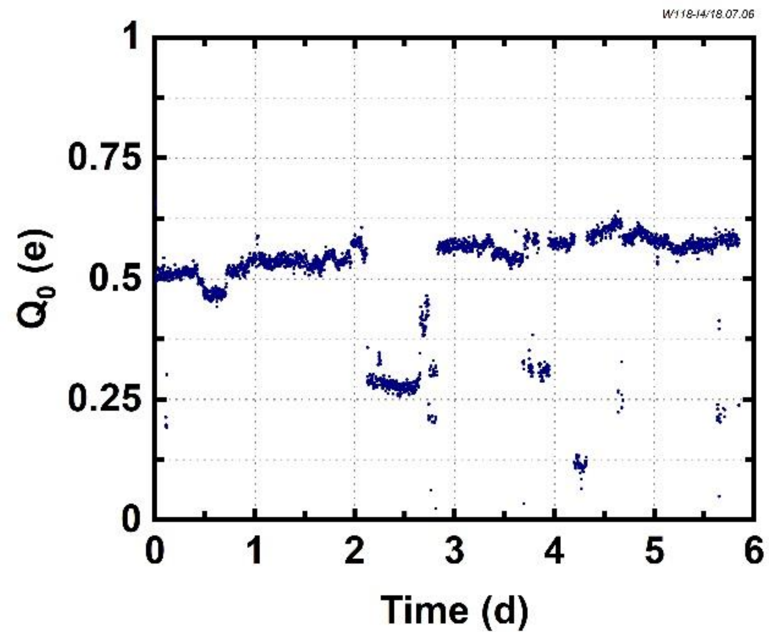

(b)

Fig. S3 Plasma oxidized AlO_x tunnel barriers implemented in Co/AlO_x/Co SETs. (a) Long-term repeating CBO taken over ≈ 5.5 days as a function of time (ΔV_g = 22.81 mV ± 0.08 mV). There are more jumps over the measurement course than the above Al/AlO_x/Al SETs, but are long periods where the phase is stable. (b) Extracted charge offset, Q_0, as a function of time using Sine method. ΔQ_0 = (0.64 ± 0.007) e. A small linear drift (0.013 e/d ± 0.0002 e/d) exists in the main trend excluding the abrupt jumps.
